# Characterizing ecosystem phenological diversity and its macroecology with snow cover phenology

**DOI:** 10.1038/s41598-019-51602-1

**Published:** 2019-10-21

**Authors:** Yi Lin, Juha Hyyppä

**Affiliations:** 1 School of Earth and Space Sciences, Peking University, Beijing, 100871 China; 2 Finnish Geospatial Research Institute, Masala, 02430 Finland

**Keywords:** Biodiversity, Phenology, Ecosystem ecology, Macroecology

## Abstract

One critical challenge of exploring flora phenology is on characterizing ecosystem phenological diversity (EPD), and thus how EPD’s performance is influenced by climate changes has also been an open macro-ecological question. To fill these two gaps, we proposed an innovative method for reflecting EPD, by taking the advantage of the often-classified inverse factor of spatial resolution discrepancy between the used remote sensing datasets of vegetation phenological dates (green-up and brown-up) and snow cover phenological dates (SPDs) (onset and end) around the Arctic, and further, we examined the cross response/feedbacks of the two kinds of EPDs to the two categories of SPDs. We found that the circumpolar green-up and brown-up EPDs both were shrinking, driven more by the delaying of the onset SPDs than the advancing of the end SPDs; North America and North Eurasia performed with inconsistent EPD response/feedbacks to the related SPD anomalies; and further, the EPD-SPD response/feedbacks in some locations exhibited the time-lag effect, e.g., the green-up EPDs made the strongest response to the onset SPDs of two years earlier. Overall, the validated method and the new findings are of implications for improving the phenology modules in Earth system models, and the contributions of the present study have enlightening significance for kicking off the new EPD branch in macrosystem phenological ecology.

## Introduction

Following phenophase^1^ and phenological shift^2^, phenological diversity that is defined as the variety of phenological dates of life^3^ now is turning into another ‘hot-spot’ phenological trait, highlighted by the communities concerning biotic phenology^3–5^. Examining the performance, feature, and evolution of phenological diversity, as an important indicator of biodiversity^6^, is of extensive significance for understanding of how species, communities, and ecosystems may make response/feedbacks to climate changes^7^. To characterize this phenological trait, people proposed specific parameters such as phenological synchrony^4^ or asynchrony^5^, which are quantified as the consistency or inconsistency between the phenological dates of varying biological functional groups^4,5^, respectively. With such parameters used as the indicators, it was discovered that the phenological differences among ecotypes are not related to small genetic differences but simply phenotypic adaptations to different climatic conditions^8^. People also realized that exploring the rules of phenological diversity offers a major avenue to advance the adaptations for ecotypes like winegrapes in agriculture to severe climate anomalies^9^. Further, it was noticed that phenological synchrony has some consequences for ecological interactions and biotic population dynamics^10^, while phenological asynchrony arising from climate warming has the high potential of affecting parasite transmission, with non-linear impacts on disease burden^11^. These pioneering studies all suggested that investigating phenological diversity is of considerable implications for biology and ecology.

**Figure 1:**
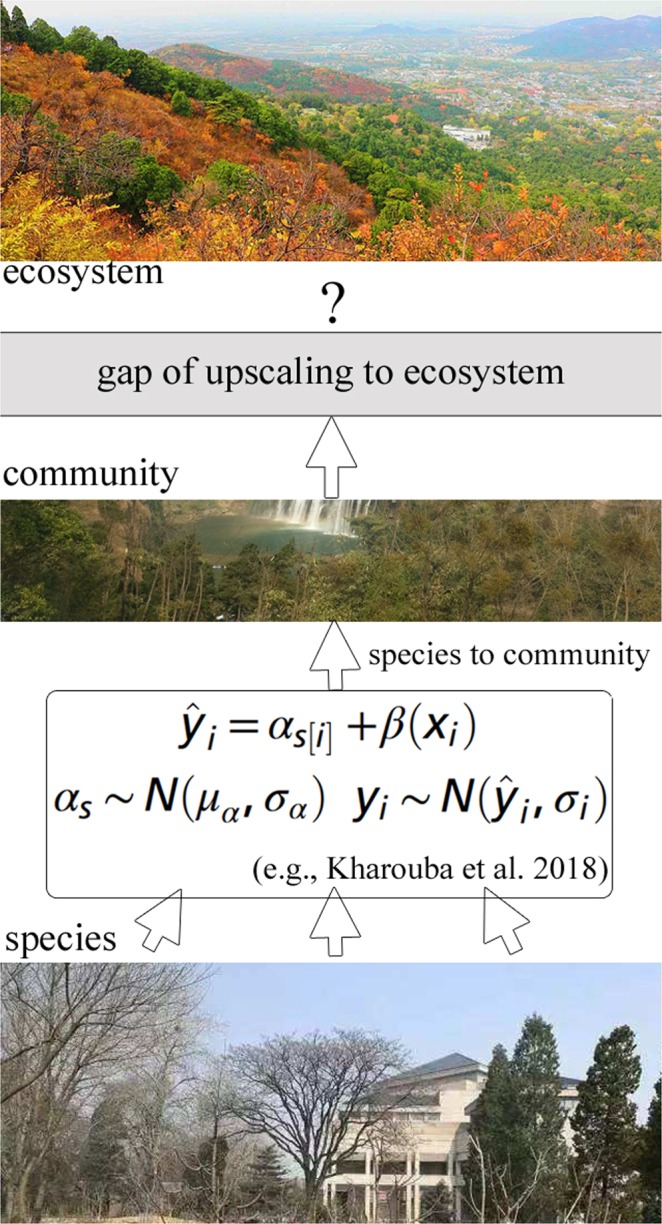
The schematic diagram of flora phenological diversity concerned by the present study, which was aimed at the of-interest gap of characterizing EPD in the currently-mainstream solution framework of upscaling from species to community, due to its complex and variable species and community compositions. The representative solution plan^7^ is presented in the bridging circle between species and community, wherein *y* is phenological or synchrony change (depending on the model), *x* is temperature change, *i* is a single iteration, *s* is species, and *ŷ* is the predicted value of *y*; the used covariate model allows variation across species in their phenological change, but estimates a single slope (e.g., the change in phenology per change in temperature) across all the species (*β*)^7^. Overall, the significance of launching this study can be intuitively learnt from this sketch map (The photographs used as the base images were taken by Yi Lin).

However, study on phenological diversity so far is still in its infancy. That is, the limited studies on phenological diversity are yet struggling at the initial phase of stepping forward from species to communities^12^. Their common scheme is to test if multiple species, hypothetically representing a community, can sustain the effect of phenological synchrony or not^13^. Some evidences from *in-situ* observations and controlling experiments alluded that maintenance of this synchrony in the context of climate changing is common^14–16^, while others demonstrated that the degrees of the changes in synchrony vary between populations^17,18^ or, at least, are larger than expected^19,20^. This contradiction has triggered a puzzle – whether shifts toward phenological asynchrony are widespread^21–23^. In other words, the evidences to date have not drawn a clear picture about how prevalent and large the shifts in phenological synchrony from species, taxonomic groups, to communities have been in response to recent climate changes^7^. In turn, this has set up almost no ecological theoretical bases for upscaling the phenological diversities of species and communities to infer the global performance of macro-scale ecosystems (macroecosystems), as marked by the gray-colored ‘broken road’ in Fig. 1. Hence, it is in intense demand to develop efficient methods for reflecting the situations of ecosystem-level phenological diversity.

Another evidence for this ‘infant’ point is that previous considerations of environmental forces on phenological synchrony were primarily restricted to temperature^24–26^. In fact, it was also found that snow is another key environmental factor capable of influencing ecosystem functions^27^. Steltzer *et al*. earlier noticed that while the timing of snowmelt in spring, at least partially, depends on spring air temperature, snowmelt and temperature often act independently from one another to control the flowering timing^28^. Iler *et al*. were also aware that some species seem to be approaching their limits of phenological shifts in response to snowmelt rather than temperature^29^. Winkler *et al*. further observed that the near-term, community-wide changes in phenology responding to the variations in snowmelt timing are likely to be contingent on the legacy of past climates on community structures^30^. These endeavors exposed more ecological roles of snow cover phenology^27^ on plant seasonal timing, but their inferences were still aimed at community levels (Fig. 1, with the mathematical scheme of the representative up-scaling method^7^ shown). So, the knowledge about the macroecological effect^31^ of snow cover phenological anomalies on ecosystem phenological diversity (EPD) is still almost void.

As pointed out in Fig. 1, a ‘big’ challenge of this field in the next stage is to improve our ability to understand such eco-effects and project the full consequences of various environmental drivers on larger-scale community- and ecosystem-level phenological diversities^7^. One of the reasons of deeming it ‘big’ is that these two levels tend to show complex phenological combinations and interactions^22^, particularly for the latter ones. This is illustrated by the fact that species-specific climate response within ecological communities proved to be capable of disrupting the synchrony of co-evolved mutualisms, which are generally based on the shared timing of seasonal bio-events^24^. This kind of disruption effects may become stronger at the ecosystem levels, investigating whose underlying ecological processes, consequently, facilitates bettering Earth system models in simulating global changes^32^. However, simultaneously collecting the datasets of phenological diversity at the species and macroecosystem scales based on those traditional *in-situ* phenological observations or small-scale experiment approaches^1,7^ is a difficult, actually almost impossible, task in practice. This, substantially, restricts quantitative assessments of EPDs from the beginning, also as the root cause of the last ‘knowledge-void’ problem.

To address these two disciplinary-foundation gaps, we creatively assumed the newly-published time-series data of plant phenological product (1999–2013)^33^ and the global data of snow cover phenological dates (SPDs) (2001–2014)^34^, together as an alternative strategy for quantitatively characterizing the EPDs (green-up and brown-up) and SPDs (onset and end) over the Northern Hemisphere (>45°N) (NH), to examine: (i) whether the Arctic circumpolar EPDs performed with some trends during the study period, and (ii) whether their developments can be attributed to the interannual SPD variations. Note that the ‘interannual’ here considered the year-long phase from snow cover onset to ecosystem brown up of the next year (OB) as one ‘integral year’ for exploring the response, while ecosystem green up to snow cover end of the next year (GE) for feedbacks. Deriving both EPDs’ and SPDs’ trends and their relationships facilitates handling more mysteries about EPD, which is critical for comprehensive predictions of future shifts in EPD due to climate changes.

## Results

### EPDs’ and SPDs’ trends

**Figure 2:**
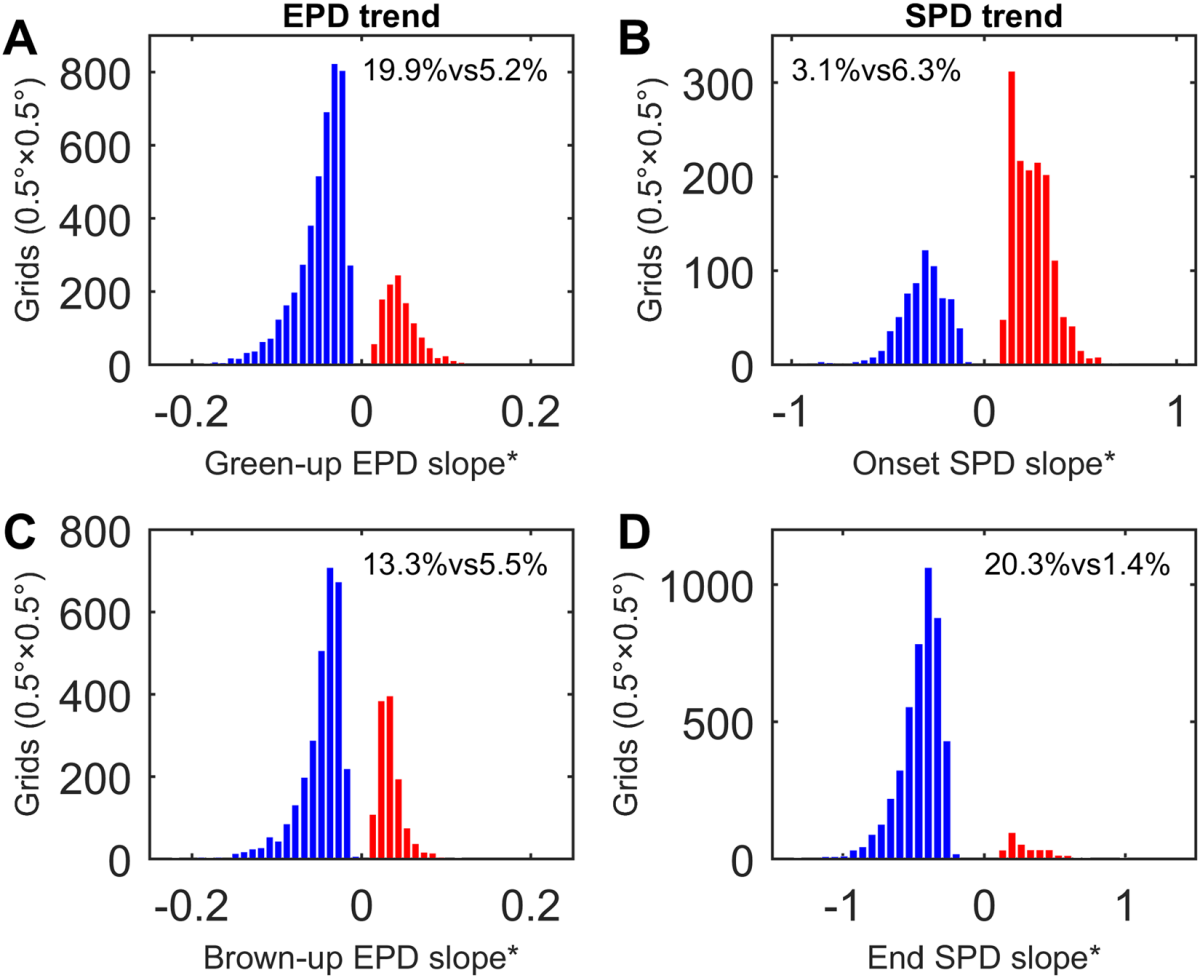
Histograms of the (**A**) green-up and (**C**) brown-up EPD trends and the (**B**) onset and (**D**) end SPD trends over the NH, in terms of their fitted slopes along with the years increasing, all calculated at the significance level of p < 0.1 (indicated by *). The bars of the negative and positive trends are blue- and red-colored, with their area ratios (formatted as B% and R%, respectively) compared to the NH listed in the form of B%vsR%.

The two histograms of the statistically significant green-up and brown-up EPD trends (1999–2013) over the NH (SI Appendix, Fig. S1A,B, respectively) were derived (Fig. 2A,C). For the whole NH, these key EPD features both seemed to be shrinking (the average green-up EPD trend = −0.21 days/decade and the average brown-up EPD trend = −0.17 days/decade). For more details, the histograms of those statistically significant green-up and brown-up EPD trends over the North America (>45°N) (NA) (SI Appendix, Fig. S2A,C) and North Eurasia (>45°N) (NE) (SI Appendix, Fig. S2B,D) separately were also derived. For the NE, the degradations in green-up and brown-up EPDs were both stronger (the average green-up EPD trend = −0.26 days/decade and the average brown-up EPD trend = −0.21 days/decade) than those of the NH; but for the NA, its degradation in brown-up EPD was weaker, even negligible (the average brown-up EPD trend = −0.02 days/decade), while its green-up EPD development was in a totally negligible but still positive tendency (the average green-up EPD trend = 0.0001 days/decade). In summary, no obvious decreasing or increasing in EPD over the NA was observed, but an apparent decreasing in EPD over the NE was detected.

Figure 2B,D list the histograms of the statistically significant onset and end SPD trends (2001–2014) derived over the NH (SI Appendix, Fig. S3A,B, respectively). For the whole NH, these two typical SPD features (the average onset SPD trend = 0.80 days/decade and the average end SPD trend = −3.89 days/decade) seemed to be getting closer. That is, in a whole sense the circumpolar snow cover onsets were delaying and the snow cover ends were advancing, somehow like the Arctic circumpolar having ‘shorter breaths’ in interannual snow cover temporal length. Then, the histograms of the statistically significant onset and end SPD trends over the NA (SI Appendix, Fig. S4A and Fig. S4C) and NE (SI Appendix, Fig. S4B,D) were also determined. For the NE, its delaying in onset SPD and advancing in end SPD (the average onset SPD trend = 0.60 days/decade and the average end SPD trend = −3.99 days/decade, respectively) were approximate to the whole NH, with a little slighter delaying in snow cover onset and a little stronger advancing in snow cover end. For the NA, its advancing in end SPD was weaker but comparable to the NH (the average end SPD trend = −3.48 days/decade), but its delaying in onset SPD was more serious (the average onset SPD trend = 2.57 days/decade). The derived trends in the study period might be different from what they were in the whole last century^35^, but totally, obvious delaying in onset SPD and advancing in end SPD were discovered over both of the NA and NE.

### EPDs’ response/feedbacks to SPDs

**Figure 3:**
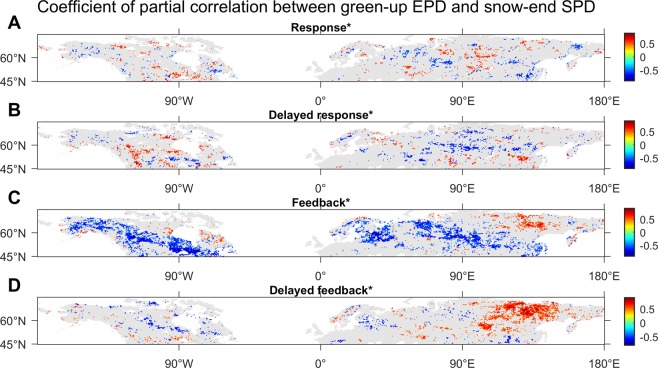
Spatial distributions of the optimal coefficients of correlations, in the cases of probing into (**A**) response, (**B**) delayed response, (**C**) feedback, and (**D**) delayed feedback respectively, between the green-up EPDs and end SPDs over the Northern Hemisphere (>45°N) in the study period, all calculated at the significance level of p < 0.1 (indicated by *). The range of the derived correlation coefficients relating to the case of (**A**) response is from −0.90 to 0.89, the range of the derived correlation coefficients relating to the case of (**B**) delayed response is from −0.89 to 0.90, the range of the derived correlation coefficients relating to the case of (**C**) feedback is from −0.91 to 0.91, and the range of the derived correlation coefficients relating to the case of (**D**) delayed feedback is from −0.80 to 0.90.

**Figure 4:**
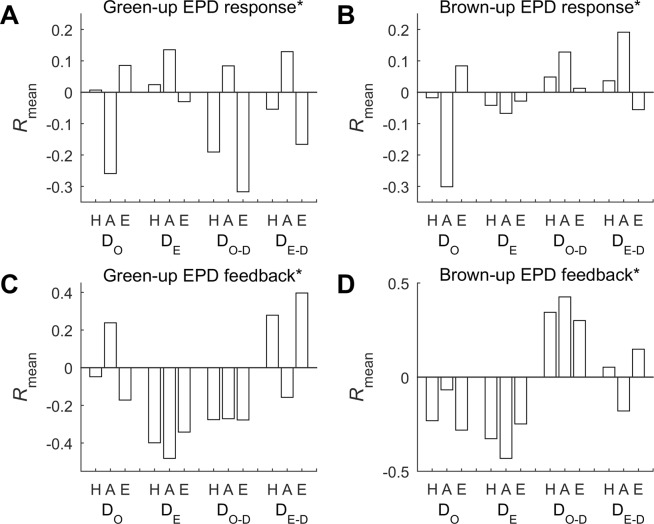
Bars of the mean coefficients of the optimal correlations (*R*_mean_) for characterizing the (**A**) green-up and (**B**) brown-up EPDs’ response and (**C**) green-up and (**D**) brown-up EPDs’ feedbacks to the onset and end SPDs in the within-the-same-OB-year and within-the-same-GE-year manner (D_O_ and D_E_) or in the one-OB-year-delayed and one-GE-year-delayed manner (D_O-D_ and D_E-D_, respectively), all calculated at the significance level of p < 0.1 (indicated by *), in terms of the NH (H), NA (A), and NE (E) as listed in the labels of the X axis.

The simultaneous statistics of the responses and the delayed responses of the green-up EPDs to the onset SPDs (SI Appendix, Fig. S5A,B) and end SPDs (Fig. 3A,B) over the NH, NA, and NE were derived (Fig. 4A). In terms of *R*_mean_ (the mean of the coefficients of the statistically-significant correlations calculated by following the method in sub-Section ‘Analyses’), it could be inferred that the circumpolar green-up EPDs (>45°N) were briefly affected negatively by snow cover onsets in a one-OB-year-delayed way (denoted as D_O-D_, hereafter). The responses over the NE worked in the same pattern (D_O-D_, with the subscripts ‘-D’ marked to indicate the time-lag effect hereafter), whereas the EPDs over the NA dominantly were influenced negatively by snow cover onsets in a within-the-same-OB-year way (D_O_).

Figure 4B shows the statistics of the responses and the delayed responses of the brown-up EPDs to the onset SPDs (SI Appendix, Fig. S6A,B) and end SPDs (SI Appendix, Fig. S7A,B) over the NH, NA, and NE. By following *R*_mean_, we found that the brown-up EPDs over the whole NH were driven positively by snow cover onsets in a one-OB-year-delayed way (D_O-D_). However, neither the NA nor the NE acted in the same manner. The brown-up EPDs over the NA were relatively more affected negatively by snow cover onsets, and the brown-up EPDs over the NE were more driven positively by snow cover onsets, both in a within-the-same-OB-year way (D_O_).

The synchronous statistics of the feedbacks and the delayed feedbacks of the green-up EPDs to the onset SPDs (SI Appendix, Fig. S5C,D) and end SPDs (Fig. 3C,D) over the NH, NA, and NE were generated (Fig. 4C). On account of *R*_mean_, it was concluded that the Arctic circumpolar green-up EPDs (>45°N) dominantly influenced snow cover ends negatively in a within-the-same-GE-year way (D_E_). The feedbacks over the NA behaved in the same mode (D_E_), whereas the green-up EPDs over the NE wholly exerted a positive effect on snow cover ends in a one-GE-year-delayed way (D_E-D_).

Figure 4D presents the statistics of the feedbacks and the delayed feedbacks of the brown-up EPDs to the onset SPDs (SI Appendix, Fig. S6C,D) and end SPDs (SI Appendix, Fig. S7C,D) over the NH, NA, and NE. Based on the index *R*_mean_, it was derived that the Arctic circumpolar brown-up EPDs (>45°N) primarily made a positive feedback to snow cover onsets in a one-GE-year-delayed way (D_O-D_). The dominant feedbacks over the NE behaved similarly, but the brown-up EPDs over the NA dominantly interfered with snow cover ends negatively in a within-the-same-GE-year way (D_E_).

The specific parameters relating to all of the above-listed cases, performing with the optimal correlations statistically, are listed in Table 1. For green-up EPD’s response and brown-up EPD’s feedbacks, the NE acted like the NH, while the NA did the best with other SPDs in different ways. For green-up EPD’s feedbacks, the NA acted more like the NH, also in terms of the ratio between the covers of negative and positive trends. For brown-up EPD’s response, neither the NA nor the NE acted similarly as the NH. Moreover, the findings about the distinctive area ratios between NA and NE for some EPD-SPD response/feedback cases project a new challenge following this study, i.e., what is the mechanism of ecosystem-snow interactions causing such differences.

**Table 1:** Statistics of the optimal cases of correlations for characterizing the EPDs’ response/feedbacks to the SPDs over the NH, NA, and NE, and their area proportions compared to the corresponding continents (>45°N), separately for the negative and positive correlation coefficients (*R*) derived at the significance level of p < 0.1 (indicated by *).

EPD vs. SPD	The Northern Hemisphere (>45°N)			North America (>45°N)			North Eurasia (>45°N)		
	snow trait	area proportion (%)		snow trait	area proportion (%)		snow trait	area proportion (%)	
	optimal SPD*	negative R*	positive R*	optimal SPD*	negative R*	positive R*	optimal SPD*	negative R*	positive R*
Green-up EPD response	D_O-D_	7.04	3.86	D_O_	6.08	2.44	D_O-D_	8.27	2.79
Brown-up EPD response	D_O-D_	4.20	4.97	D_O_	6.73	2.29	D_O_	5.17	6.99
Green-up EPD feedback	D_E_	17.41	3.62	D_E_	23.49	2.82	D_E-D_	2.28	11.68
Brown-up EPD feedback	D_O-D_	3.69	13.29	D_E_	12.74	1.79	D_O-D_	4.17	12.26

Further, the cases with the subscripts ‘-D’ indicate that the time-lag effect existed between EPDs and SPDs in their response/feedbacks. For example, the green-up EPDs showed the strongest response to the onset SPDs of two years earlier in some locations, and this has validated the hypothesis of the time-lag effect existing. The specific situations in details would become more complicated when the scattered spatial distributions of the grids with the best correlations derived were examined (Fig. 3; SI Appendix, Figs S5–S7).

Overall, the green-up and brown-up EPDs for the NH, NA, and NE were shrinking in a whole sense, and this was triggered more by the delaying of snow cover onsets, instead of the advancing of snow cover ends; the green-up EPDs over the NH, NA, and NE consistently implemented their feedbacks more closely to the end SPDs in the last decade, whereas the brown-up EPDs performed with divergent feedbacks to either of the SPDs; finally, the EPD-SPD response/feedbacks in some cases also exhibited the time-lag effect, e.g., the green-up EPDs showing the strongest response to the onset SPDs of two years earlier.

## Discussion

Compared to the currently-mainstreamed researches on attempting to attribute flora phenological changes to long-term temperature trends based on meta-analyses^7^ of species-level observations for deriving community-level phenologicial knowledge^12–16^, this study directly concentrating on macrosystem phenological ecology by innovatively using the derivations from different remote sensing data^33,34^ is relatively rare, particularly accounting for snow cover phenology^27^ as another essential climate force^4,28,30^. Those macroecological findings can help to fill the gap (Fig. 1) toward understanding of how climate changes influence Arctic circumpolar ecosystem phenology and how the changes of ecosystem phenological traits adjust the modes of climate developments. Overall, this work has opened an enlightening way for studying the EPD branch of macrosystem phenological ecology.

As previous studies showed that the difficulty in attributing changes in phenological synchrony to temperature change can be a function of both methodology and biology^7^, our study mirrored this difficulty from the same two aspects as well. In methodology, the applied means of correlation analysis is still featured with uncertainties, as illustrated by the inconsistency between the derived SPD traits over the NH, NA, and NE (Table 1). Generally, the EPD change and response/feedback performance for the whole NH shall follow the mode of NA or NE that plays a leading role, but an exceptional case exists (see the row of ‘brown-up SPD response’ in Table 1). This inconsistency presents the shortage of the assumed method when it meets the extreme scenario with approximate area proportions for the positive and negative correlation cases. In light of the suspect that the high uncertainty might be briefly related to the short temporal length of the time series of the analyzed data^7^, the authors suggested to develop more fundamental EPD data of longer time series than the used one for retrieving more reliable EPD values and reducing the uncertainties.

In biology, the complexity in the processes of snow variations influencing EPD trends is also cued by the results. The cases of the derived statistically significant EPD trends approaching 0 are quite few (see Fig. 2A,C), implying that the interannual variations of green-up and brown-up EPD values were intense. This suggested that apart from the interannual variations of snow cover timing, EPD may also be affected by other environmental factors. In fact, even for the snow factor, it may be affected by other environmental factors^36^, as evidenced by the similar scenarios that few cases of statistically significant snow phenophase trends approaching 0 were extracted (Fig. 2B,D). Substantially, it is challenging to link EPD variations directly to SPD changes because snow is a complex phenological cue^37^. In the case of forest ecosystems, snow cover can affect land surface temperature, solar irradiance, and evapotranspiration, and these environmental factors can also reversely reshape the morphology of snow cover distribution, all together regulating the timing and magnitude of phenological shifts^38,39^. These all suggest that more comprehensive analyses, with the potential environmental factors^40^ as many as possible considered, need to be carried out later.

Although the used methods take on the above-listed uncertainties, this study is of fundamental implications for filling the targeted gaps between ecosystem-level phenological developments and climate changes and further projecting the future of biosphere under global change. In other words, although the underlying mechanism of species and communities behaving with such global EPD performance after adapting to environmental changes is unclear, the EPD-SPD response/feedback rules statistically derived in this study can cue a new framework of Arctic circumpolar ecosystem processes involving inter-annual phenological variations of snow cover accumulations to improve the performance of phenology modules in current Earth system models^27^.

In fact, it is a so difficult task to investigate the underlying mechanism of SPD influencing EPD, which, substantially, relates to an integration of complex macro-ecological interactions rather than a plus of traditional physiological responses^30,39^. As the previous studies told, the changes in phenological diversity can have fitness consequences^41^ and affect ecosystem-level properties like primary productivity^42^ and pollination^43^, but such consequences do not always emerge^44^ or seem to be scale-dependent^45^. It is also unclear the extent to which species will be able to evolutionarily adapt to restoring phenolgical synchrony^46^. This means that it is inappropriate to give some simple hypothetic explanations to the derived rules. Thereby, the next challenge in this direction is to further handle the gap between the remote sensing-based inferences on EPD changes at the regional/continental scales and the previous findings about phenological diversity performance derived by upscaling from species to communities^12–16^.

In summary, an innovative method was proposed for breaking through the conventional gap of characterizing ecosystem phenological diversity. The validated method and those macroecological findings are of implications for improving the phenology modules in various Earth system models, and the contributions in this study can mean a new era of the novel EPD branch in macrosystem phenological ecology.

## Methods

The rationale of operating this study was directly using the remote sensing-derived datasets in macrosystems ecology^47^ to handle the basic challenges long faced by traditional phenological ecology. The proposed method of characterizing EPD was based on the discrepancy between the spatial resolutions of the two used datasets^33,34^. As we know, snow cover onsets (ends) tend to simultaneously cover massive regions^27^, while ecosystem green-up (brown-up) dates^48^ are distinctive for different communities and species at finer scales^7^. The 0.5° spatial resolution of the snow dataset^34^ can reflect the trait of SPDs in the former scenario, while the 4 km (~0.0357°) resolution of the plant phenological date product^33^ can mirror the feature of flora phenological dates in the latter one. Given that one grid in the SPD data^34^ corresponds to ~14 × 14 grids in the EPD-source data^33^, the EPD characterization was operated by unifying the resolution of the EPD-source data to the SPD data, with each grid of the derived EPD data defined as the standard deviation of phenological dates within its corresponding 14 × 14 grids, analogous to the definition of phenological synchrony^4^ and asynchrony^5^. Then, their linear correlations with the effect of characterizing the EPD-SPD response/feedbacks were examined, in the within-the-same-OB-year, one-OB-year-delayed, within-the-same-GE-year, and one-GE-year-delayed manner, respectively. This innovative remote sensing-based solution strategy is theoretically reasonable, with the high potentials of handling the problem of EPD data shortage that lead to the ‘big challenges’ targeted in this study.

### EPD data

The EPD-source data was the Arctic circumpolar vegetation dynamics product for global change study^33^. Specifically, based on the SPOT VGT data, Gonsamo and Chen derived the first set of phenology index-based plant dynamics product, comprising green-up and brown-up phenological dates, for the NH during 1999–2013^33^. With the factors like snow^27^ possibly influencing the derivation of the phenological dates considered, the spatial resolution of the product was unified into 4 × 4 km (0.03571428 × 0.03571428°). The validation based on the data from the flux tower sites of deciduous broadleaf forests, evergreen needle-leaf forests, mixed forests, and wetlands over the NA and NE suggested good agreements between the phenological dates from this Arctic circumpolar vegetation dynamics data and the reliable estimates from CO_2_ flux measurements. The validation proved that the circumpolar vegetation dynamics product is an improvement over the operational global MODIS Combined Land Cover Dynamics MCD12Q2 product for the Arctic circumpolar region^33^. For derivation of the EPDs required in this study, the standard deviations of the phenological start and end dates for the matrix of 14 × 14 grids^33^ corresponding to any grid in the SPD dataset^34^ were calculated (Eq. 1) to characterize the green-up and brown-up EPDs, respectively.

1 $${v}_{{\rm{EPD}}}^{i}=\sqrt{\frac{1}{196}\mathop{\sum }\limits_{j=1}^{14}\mathop{\sum }\limits_{k=1}^{14}{({x}_{j,k}^{i}-\overline{{x}^{i}})}^{2}}$$

where $\overline{{x}^{i}}=\frac{1}{196}\mathop{\sum }\limits_{j=1}^{14}\mathop{\sum }\limits_{k=1}^{14}{x}_{j,k}^{i}$, and ${v}_{{\rm{EPD}}}^{i}$ denotes the *i* th SPD grid-related EPD value, derived based on the corresponding phenological date ${x}_{j,k}^{i}$ set of 14 × 14 grids. Compared to the ranges of the start and end values within each matrix^33^, assuming standard deviation can theoretically avoid the influences of the outlier values in some 4 × 4 km grids and hence can better quantify the phenological diversities at the ecosystem scales. The theoretical foundation of proposing such a measure is that the used EPD-source data^33^, substantially, is an EPD product at the finer scale.

### SPD data

The SPD data was extracted from the snow seasonal timing data in the northern middle and high latitudes (2001–2014)^34^. This data is an improved merge of five extensively-used snow datasets, including the reanalyzed dataset of daily snow depths generated by the Canada Meteorological Center, the binary daily snow cover mask derived from both the Interactive Multi-sensor Snow and Ice Mapping System and the Northern Hemisphere Weekly Snow Cover and Sea Ice Extent, the 8-Day Level 3 snow cover fraction product derived from the MODIS data, and the snow water equivalent data derived from the Near-real-time Ice and Snow Extent data. A multi-data approach was employed in developing the combined snow cover phenology matrix that can integrate snow cover timing information probed from multiple sources of snow observations. In this data, the snow cover accumulation season is defined to be from November of previous year to February of current year, and the snow cover melting season is from March to June of current year^34^. For daily snow observations, the snow cover onset date is defined as the first five consecutive days on which snow was observed to cover the ground surface in accumulation season, and the snow cover end date is defined as the last five consecutive days when snow cover was noticed in the melting season^34^. Specifically for the derivation of the SPDs used in this study, the Arctic circumpolar region higher than 45°N in latitude was simply extracted from the SPD-source data.

### Analyses

This study used partial-correlation analysis to probe the response/feedback effects of the green-up and brown-up EPDs to the onset and end SPDs around the Arctic. This approach can exclude the confounding effect of other climate variables (e.g., rainfall, temperature, and solar radiation) and the covariate effect between snow cover onsets and ends^32^. Specifically, the correlation degrees between the derived EPDs (green-up and brown-up) and SPDs (onset and end) were explored, and further, the explorations were extended to consider the effects of delayed response/feedbacks. To characterize this time-lag effect, the year-long period briefly from snow cover onset to brown-up of the next year as one ‘effective’ OB year was defined for investigating the effect of response, and the year-long period briefly from green-up to snow cover end of the next year as one ‘effective’ GE year was defined for probing the feedback effect. The correlations between the EPDs and SPDs both from the same OB year were classified as exploring the response in the within-the-same-OB-year way, and the correlations between the EPDs and the SPDs from the earlier OB year as investigating the response in the one-OB-year-delayed way; the correlations between the EPDs and SPDs both from the same GE year as examining the feedbacks in the within-the-same-GE-year way, and the correlations between the EPDs and the SPDs from the later GE year as probing the potential feedbacks in the one-GE-year-delayed way. The latter scenarios in these response/feedback explorations could reflect the time-lag effect. Since the checking of the time-lag effect was operated onto two explicit time series of the used datasets, the autocorrelation phenomenon often met in exploring the relationships between the time series of multiple kinds of features^27^ could be neglected here. Note that in subject to the short term of the assumed EPD and SPD data, only the effect of one year delaying was taken into account. Correspondingly, all of the partial correlation analyses were implemented at the significance level of p < 0.1 (indicated by *) in order to extract more grids with distinguishable response/feedbacks.

## Supplementary information

Supplementary information
