# Supplementary material for: Characterizing ecosystem phenological diversity and its macroecology with snow cover phenology
Source: Sci Rep. 2019 Oct 21;9:15074. doi: 10.1038/s41598-019-51602-1 (PMC6803678; doi:10.1038/s41598-019-51602-1)
Supplement: Supplementary file 1 — Supplementary information [file 41598_2019_51602_MOESM1_ESM.pdf]

Supplementary Information

Characterizing ecosystem phenological diversity and its macroecology with snow cover phenology

Running Head: Characterizing ecosystem phenological diversity

Yi Lin [1,*], Juha Hyyppä [2]

[1] School of Earth and Space Sciences, Peking University, Beijing 100871, China; [2] Finnish Geospatial Research Institute, Masala 02430, Finland; [*] Correspondence: yi.lin@pku.edu.cn

Supplementary Figures

Fig. S1 – Fig. S7

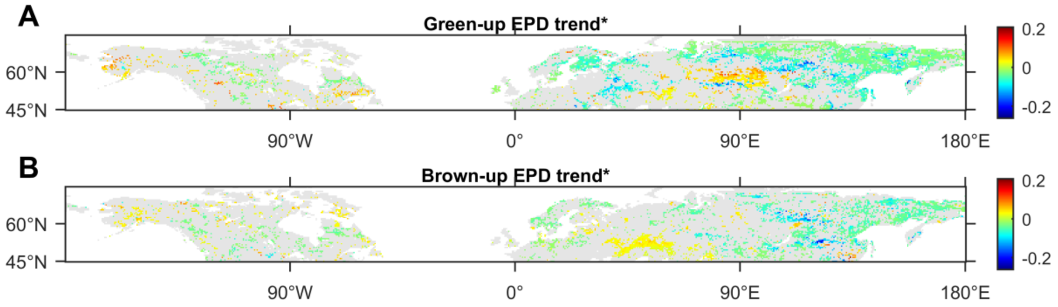

Fig. S1 Spatial distributions of the (A) green-up and (B) brown-up ecosystem-level phenological diversity (EPD) trends over the Northern Hemisphere (>45°N) during 1999–2013, all calculated at the significance level of $p<0.1$ (indicated by *). The range of the derived green-up EPD trends is from -0.26 to 0.21 days/year, and the range of the derived brown-up EPD trends is from -0.22 to 0.28 days/year.

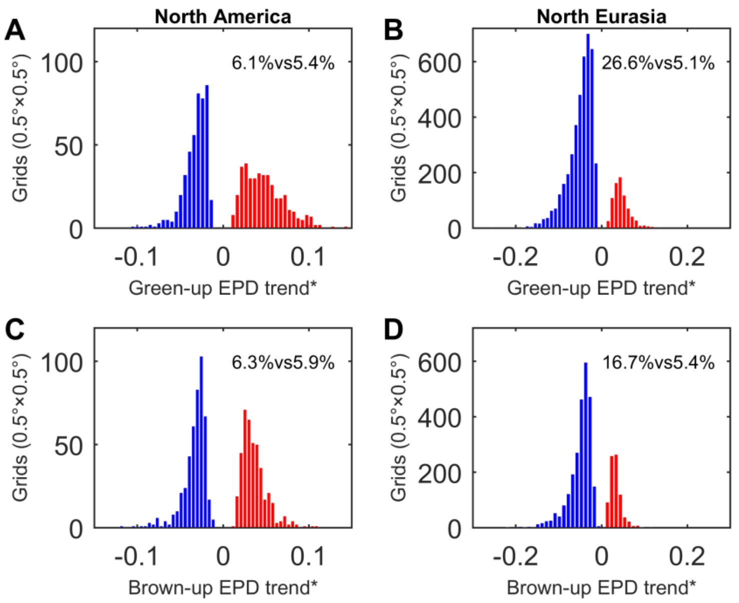

Fig. S2 Histograms of the (A) green-up and (C) brown-up EPD trends over the North America (>45°N) and the (B) green-up and (D) brown-up EPD trends over the North Eurasia (>45°N) during 1999–2013, in terms of their fitted slopes along with the years increasing, all calculated at the significance level of $p<0.1$ (indicated by *). In each sub-figure, the area proportions of the 0.5 × 0.5° grids with negative (blue-colored, quantified as B%) and positive (red-colored, R%) trends compared to the continent (>45°N) are listed, in the form of B%vsR%, respectively. The average green-up EPD trend over the North America (>45°N) is 0.0001 days/decade, the average green-up EPD trend over the North Eurasia (>45°N) is -0.02 days/decade, the average brown-up EPD trend over the North America (>45°N) is -0.26 days/decade, and the average brown-up EPD trend over the North Eurasia (>45°N) is -0.21 days/decade.

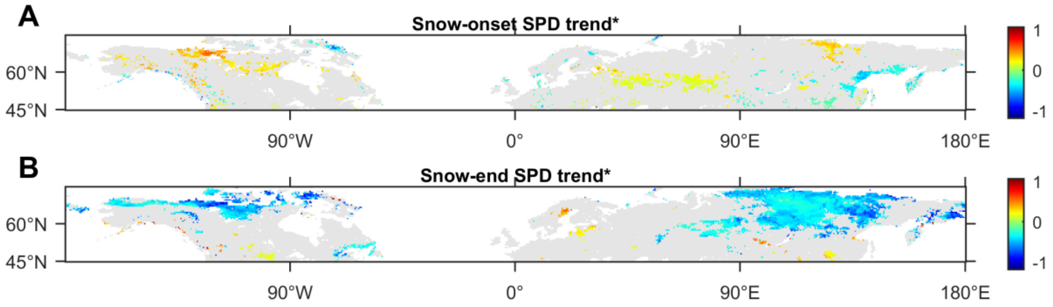

Fig. S3 Spatial distributions of the (A) onset and (B) end snow cover phenological date (SPD) trends over the Northern Hemisphere (>45°N) during 2001–2014, all calculated at the significance level of $p<0.1$ (indicated by *). The range of the derived onset SPD trends is from -1.18 to 1.06 days/year, and the range of the derived end SPD trends is from -1.88 to 1.42 days/year.

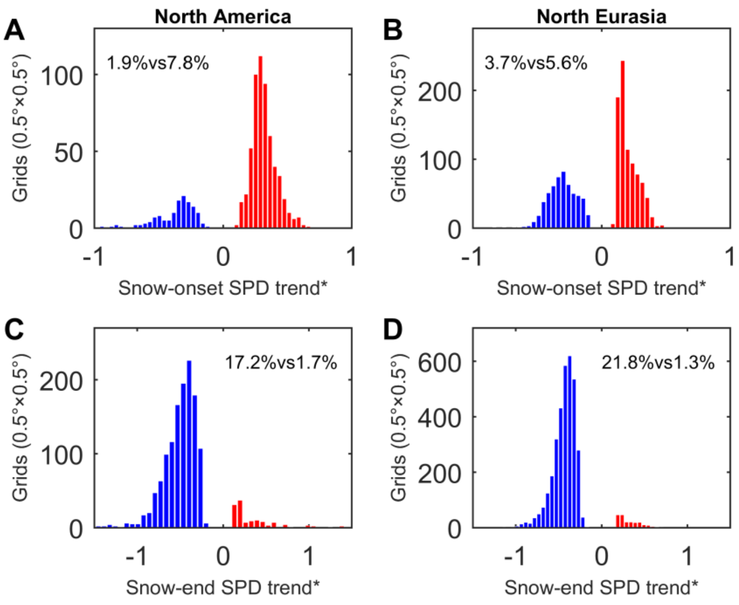

Fig. S4 Histograms of the (A) onset and (C) end SPD trends over the North America (>45°N) and the (B) onset and (D) end SPD trends over the North Eurasia (>45°N) during 2001–2014, in terms of their fitted slopes along with the years increasing, all calculated at the significance level of $p<0.1$ (indicated by *). In each sub-figure, the area proportions of the 0.5 × 0.5° grids showing negative (blue-colored, quantified as B%) and positive (red-colored, R%) trends compared to the continent (>45°N) are listed, in the form of B%vsR%, respectively. The average onset SPD trend over the North America (>45°N) is 2.57 days/decade, the average onset SPD trend over the North Eurasia (>45°N) is 0.60 days/decade, the average end SPD trend over the North America (>45°N) is -3.48 days/decade, and the average end SPD trend over the North Eurasia (>45°N) is -3.99 days/decade.

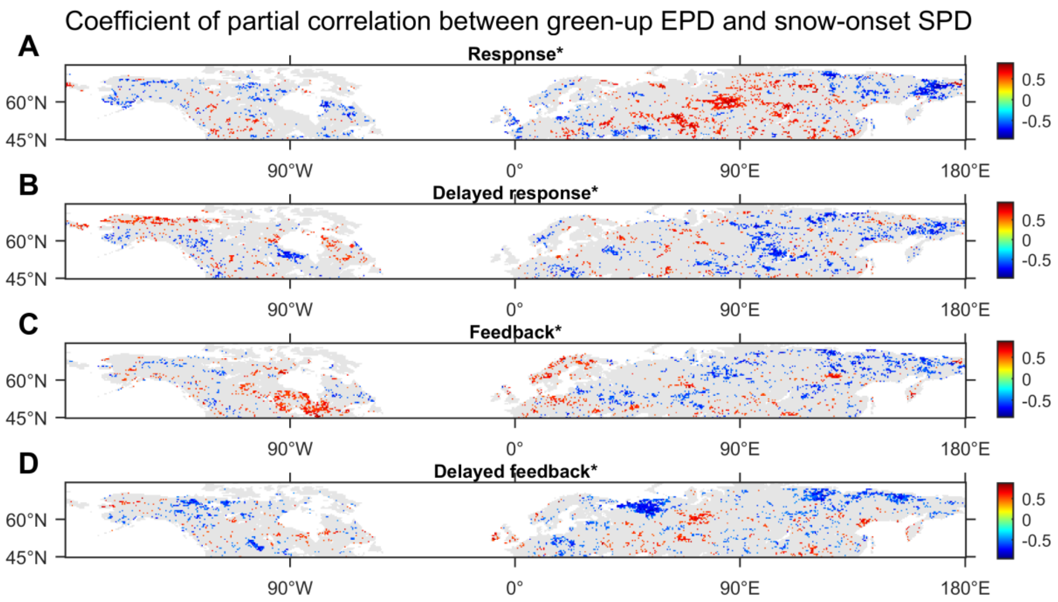

Fig. S5 Spatial distributions of the optimal coefficients of correlations, in the cases of probing into (A) response, (B) delayed response, (C) feedback, and (D) delayed feedback respectively, between the green-up EPDs and onset SPDs over the Northern Hemisphere (>45°N) in the study period, all calculated at the significance level of $p<0.1$ (indicated by *). The range of the derived correlation coefficients relating to the case of (A) response is from -0.94 to 0.88, the range of the derived correlation coefficients relating to the case of (B) delayed response is from -0.97 to 0.94, the range of correlation coefficients relating to the case of (C) feedback is from -0.90 to 0.87, and the range of correlation coefficients relating to the case of (D) delayed feedback is from -0.96 to 0.87.

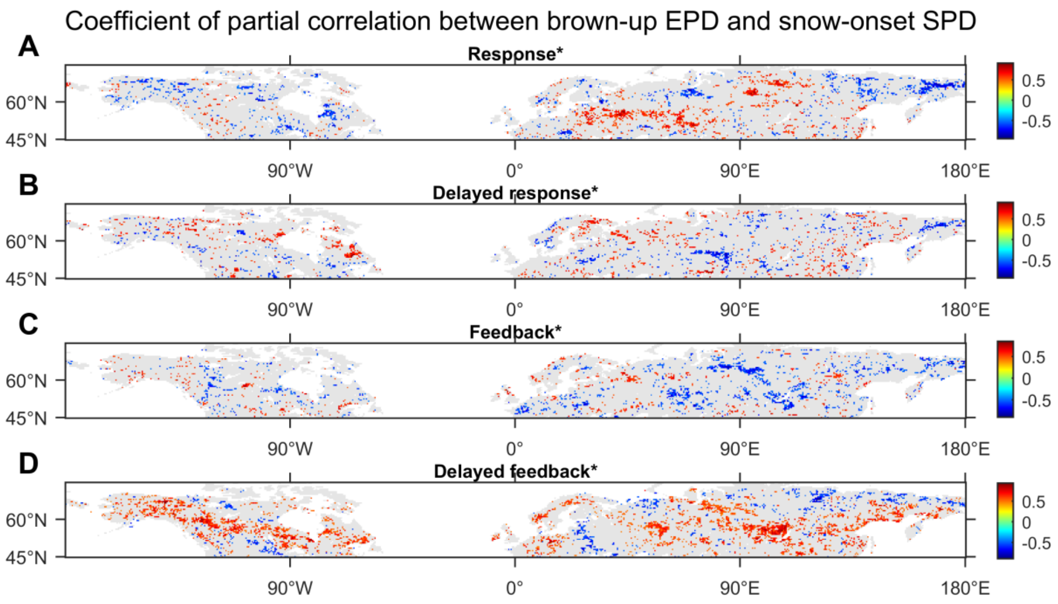

Fig. S6 Spatial distributions of the optimal coefficients of correlations, in the cases of probing into (A) response, (B) delayed response, (C) feedback, and (D) delayed feedback respectively, between the brown-up EPDs and onset SPDs over the Northern Hemisphere (>45°N) in the study period, all calculated at the significance level of p<0.1 (indicated by *). The range of the derived correlation coefficients relating to the case of (A) response is from -0.95 to 0.91, the range of the derived correlation coefficients relating to the case of (B) delayed response is from -0.93 to 0.90, the range of the derived correlation coefficients relating to the case of (C) feedback is from -0.90 to 0.85, and the range of the derived correlation coefficients relating to the case of (D) delayed feedback is from -0.89 to 0.94.

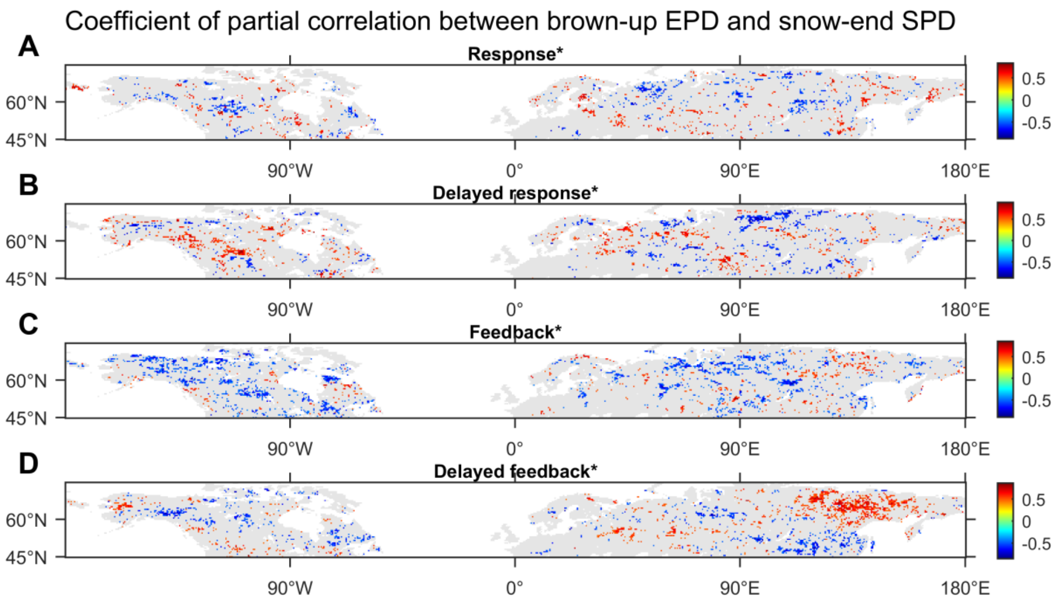

Fig. S7 Spatial distributions of the optimal coefficients of correlations, in the cases of probing into (A) response, (B) delayed response, (C) feedback, and (D) delayed feedback respectively, between the brown-up EPDs and end SPDs over the Northern Hemisphere (>45°N) in the study period, all calculated at the significance level of $p<0.1$ (indicated by *). The range of the derived correlation coefficients relating to the case of (A) response is from -0.88 to 0.83, the range of the derived correlation coefficients relating to the case of (B) delayed response is from -0.87 to 0.89, the range of the derived correlation coefficients relating to the case of (C) feedback is from -0.90 to 0.85, and the range of the derived correlation coefficients relating to the case of (D) delayed feedback is from -0.85 to 0.86.
